# d-a-Tocopheryl Polyethylene Glycol 1000 Succinate and a small-molecule Survivin suppressant synergistically induce apoptosis in SKBR3 breast cancer cells

**DOI:** 10.1038/s41598-019-50884-9

**Published:** 2019-10-07

**Authors:** Christiana M. Neophytou, Avgoustinos Mesaritis, Gregoria Gregoriou, Andreas I. Constantinou

**Affiliations:** 1 Department of Biological Sciences, Faculty of Pure and Applied Sciences, University of Cyprus, 1678 Nicosia, Cyprus; 2 Present Address: European University Research Center, Nicosia, Cyprus; 3 Present Address: University of Nicosia Medical School, Nicosia, Cyprus

**Keywords:** Breast cancer, Drug development

## Abstract

Breast cancer is the second in mortality rate malignancy among women. Despite the many advances in breast cancer treatment, there is still a need to improve drug efficacy and reduce non-specific effects. D-alpha-tocopheryl polyethylene glycol succinate (TPGS) is frequently used in the development of drug delivery systems to improve the pharmacokinetics of anti-cancer drugs and reduce multi-drug resistance. We have previously shown that TPGS not only acts as a carrier molecule but also exerts anti-cancer effects. As part of this study, we investigated the effect of TPGS with YM155, a small molecule suppressant of Survivin, in various breast cancer cell lines representing different subtypes of the disease. We aimed to evaluate the presumed synergistic effect of the TPGS-YM155 combination and reveal its mechanism of action. Our results show that the TPGS-YM155 combination acts synergistically to reduce specifically the viability of SKBR3 cells. The combination of these agents reduced activation of the AKT pathway, decreased Survivin and Bcl-2 levels, and induced caspase-dependent and independent apoptosis via the mitochondrial pathway. Importantly, the TPGS-YM155 combination did not significantly affect the viability of MCF-10A normal immortalized cells. In conclusion, the combination of YM155 and TPGS could be a promising approach against SKBR3-type breast cancer.

## Introduction

Breast cancer is the most frequently diagnosed cancer among women in Europe and the United States^1,2^. Despite the many recent advances in breast cancer monotherapy, several issues remain including severe adverse effects caused by high concentrations of chemotherapeutic drugs and acquired drug resistance. Both of these issues can be overcome or minimized by using agents that act synergistically. Since this approach requires lower concentrations of each agent, compared to monotherapy, it produces minimal adverse effects.

**Figure 1:**
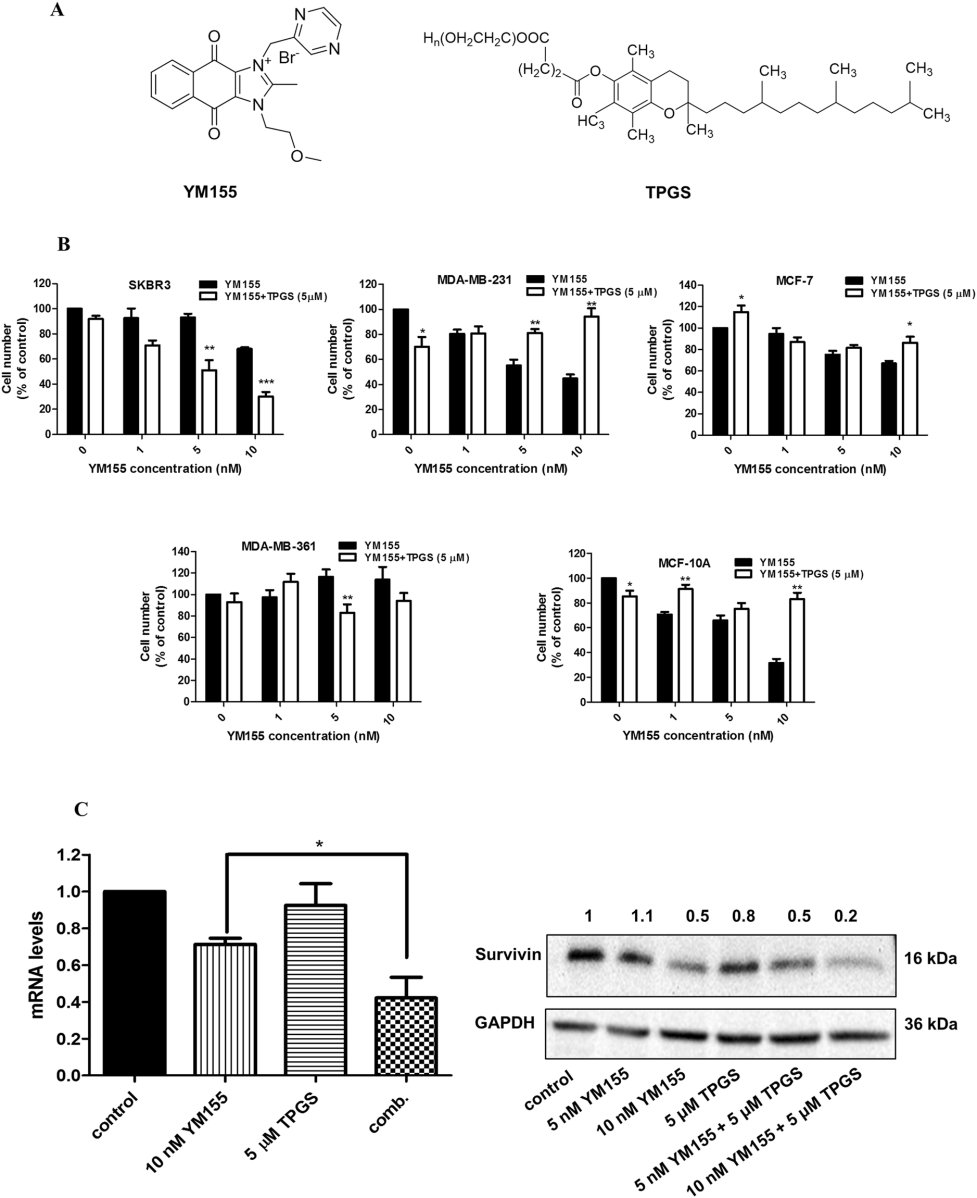
Antiproliferative effect of YM155 alone and in combination with TPGS in breast cell lines. (**A**) Chemical structures of YM155 and TPGS. (**B**) MTT assay was employed for the cytotoxicity evaluation (% cell viability) of increasing concentrations of YM155 (1, 5 and 10 nM) with or without 5 μΜ TPGS for 48 hours in SKBR3, MDA-MB-231, MCF-7, MDA-MB-361 and MCF-10A cells. IC50 of YM155 and TPGS as well the Combination Index (CI) in each cell line are shown in Table 1. (**C**) YM155 and TPGS effects on the mRNA and protein levels of Survivin at 48 hours of treatment (the blots were cropped; full length blots are shown in Fig. S6). The results represent the mean ± SEM of three different replicates and are representative of at least three different experiments. ^*^P value < 0.05, **P value < 0.01, ***P value < 0.001.

Survivin, a member of the Inhibitors of Apoptosis (IAP) family, is either undetectable or expressed at very low levels in terminally differentiated normal human tissues, but it is highly expressed in all primary tumor types^3–7^, making it an ideal target for cancer therapy. In cancer cells, Survivin acts as a multifunctional protein, implicated in the inhibition of apoptosis and the promotion of cell proliferation and angiogenesis^8,9^. YM155 (Sepantronium bromide) (Fig. 1A), is a potent small-molecule suppressant of Survivin that induces DNA damage and apoptosis in various human cancer models^10^.YM155 suppresses Survivin expression by binding to the C-terminal of RNA binding proteins interleukin enhancer-binding factor-3 (ILF3/NF110) and disrupts its binding to the Survivin promoter. YM155 has been evaluated in several clinical trials as a single agent or in combination therapy^11,12^. Even though YM155 exhibited a favorable safety/tolerability profile, it displayed modest single-agent activity in patients^13^.

D-a-tocopheryl polyethylene glycol succinate (TPGS) is a synthetic derivative of natural alpha-tocopherol, prepared from the esterification of α-TOS and polyethylene glycol (PEG) 1000 (Fig. 1A), which is frequently used in the development of drug delivery systems. The co-administration of TPGS with anti-cancer drugs improves their efficacy by enhancing their bioavailability and improving *in vivo* pharmacokinetics^14^. Combination of TPGS with other drugs leads to synergistic effects due to its ability to inhibit P-glycoprotein, an ATP-dependent drug efflux pump, also known as multidrug resistance protein 1 (MDR1) or ATP-binding cassette sub-family B member 1 (ABCB1)^15,16^. Also, as a single agent, TPGS has been found to inhibit the growth of human lung, prostate, and breast cancer cells by inducing apoptosis^17–19^.

In this study, we determined that the combination of YM155 and TPGS acted synergistically in reducing the viability of breast cancer cells. The combination of agents was effective in Her2neu-overexpressing, MDR1-wild-type SKBR3 cells but did not display synergistic effects in other breast cancer cell types or normal immortalized cells, suggesting that the mechanism of action is cell-type specific. Further mechanistic studies revealed that the compounds induce mitochondrial apoptosis via the de-activation of the AKT pathway and downregulation of Survivin. These results suggest that the markedly improved therapeutic efficacy of this combinational approach may hold significant potential for the development of future cancer treatment protocols.

## Results

### YM155 acts synergistically with TPGS to reduce the viability of SKBR3 cells

The effects of TPGS and YM155 on cell viability, alone and in combination, were tested on four human breast cancer cell lines (SKBR-3, MDA-MB-361, MCF-7 and MDA-MB-231) and one “normal” immortalized cell line (MCF-10A). All cell lines except MDA-MB-361 were sensitive to YM155 treatment (Fig. 1B and Table 1). The order of sensitivity to YM155 is as follows: MCF-7 < SKBR3 < MCF-10A < MDA-MB-231. TPGS at 5 μΜ did not significantly affect the viability of the cell lines tested, with the exception of MDA-MB-231 cells where the viability dropped to 70% (Figs 1B and S1). The combination of TPGS at 5 μΜ and YM155 at 5 and 10 nM displayed synergistic effect in reducing the viability of SKBR3 cells, with a CI index of 0.38 for the latter (Fig. 1B and Table 1). In the presence of 5 nM and 10 nM YM155 alone, the viability of SKBR3 dropped to 93% and 68% respectively, while its combination with TPGS (5 μΜ) reduced cell viability to 51% and 30%, respectively. Co-treatment of SKBR3 cells with the combination of TPGS-YM155 and with pan-caspase inhibitor z.vad.fmk or autophagy inhibitor Bafilomycine (Baf), were not able to restore cell viability (Supplementary Fig. S2) suggesting that caspase activation or autophagy are not solely responsible for the observed cell death.

**Table 1:** Cytotoxicity of TPGS and YM155 alone and in combination in breast cell lines at 48 hours.

Cell line	TPGS IC50 (μΜ)	ΥΜ155 IC50 (nΜ)	Combination Index (CI)*
SKBR3	20.77 ± 0.07	12 ± 0.09	0.38
MDA-MB-361	15.3 ± 0.04	N/A	1.37
MDA-MB-231	14.32 ± 0.08	7.26 ± 0.04	2.40
MCF-7	10.27 ± 0.03	29.30 ± 0.11	5.74
MCF-10A	57.82 ± 0.10	7.73 ± 0.02	24.10

The data are expressed as the mean (±SE) of the results from at least three separate experiments. *for combination of 10 nM YM155 and 5 μΜ TPGS.

In order to examine whether the expression of HER2 is required for the synergistic effect with YM155, we evaluated the effects of the combination on MDA-MB-361, another HER2-overexpressing breast cancer cell line. Interestingly, the combination does not affect the viability of MDA-MB-361 cells (Fig. 1B) suggesting that the presence of Her2neu alone does not ensure a synergistic effect. The characteristics of each cell line used are displayed in Supplementary Table S1.

### The combination of agents induces apoptosis in breast cancer cells

The selected agent concentrations were based on the calculated CI index as shown in Table 1. An additional consideration was to select YM155 concentrations that inhibit Survivin expression (Fig. 1C) without reducing the viability of SKBR3 cells below 50% of the control value. Based on these considerations we selected 5, 7.5 and 10 nM for YM155 and these were used for subsequent experiments.

**Figure 2:**
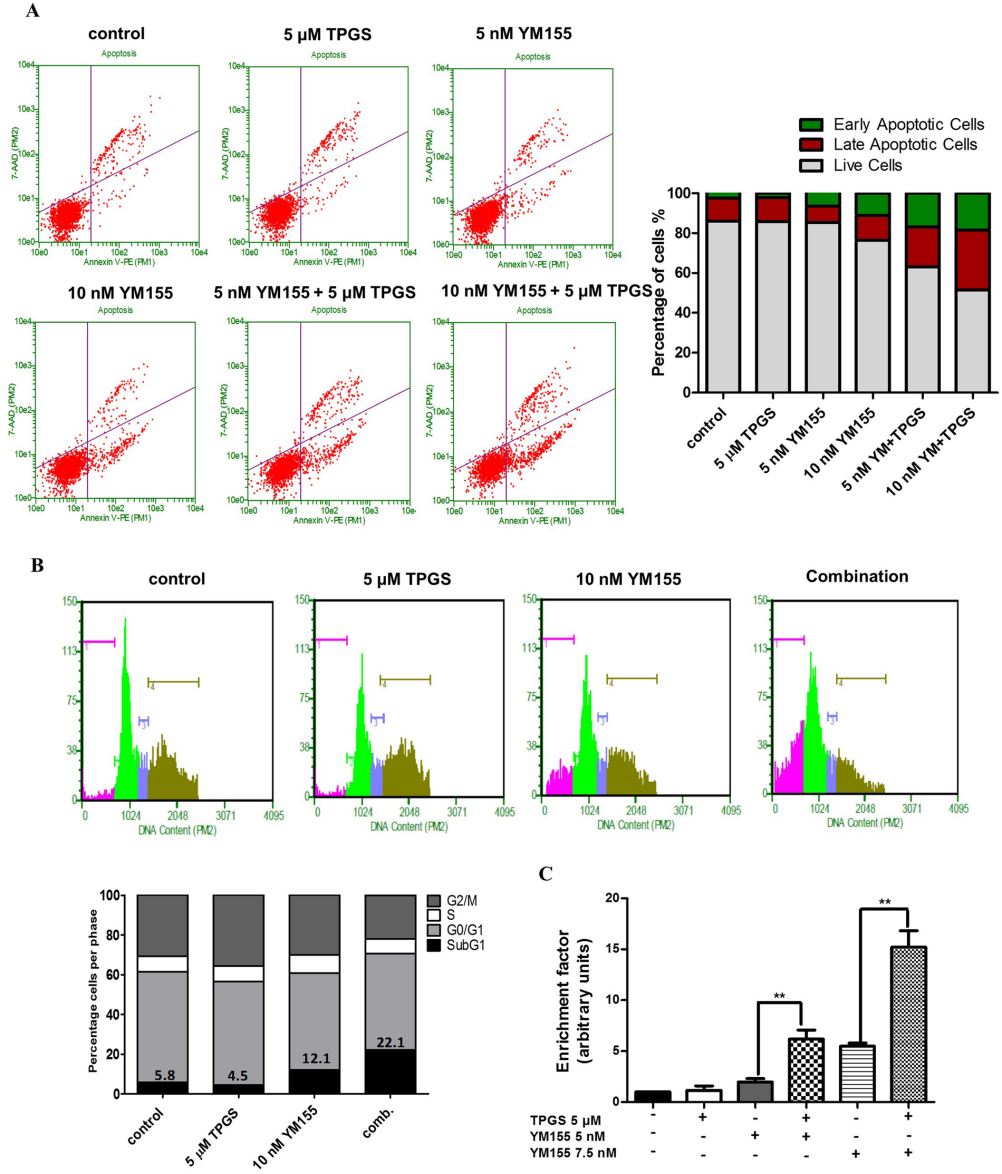
Apoptotic effect of YM155 in combination with TPGS in SKBR3 cells. (**A**) Annexin V/PI staining was employed for evaluation of the apoptotic effect (% compared to control) of increasing concentrations of 5 and 10 nM YM155 with or without 5 μΜ TPGS for 48 hours. (**B**) Cell cycle analysis following treatment with 5 μΜ TPGS and 10 nM YM155 alone or in combination for 48 hours. (**C**) Elisa-based DNA fragmentation detection in SKBR3 cells treated with 5 or 7.5 nM YM155 alone or in combination with 5 μΜ TPGS at 24 hours. The results represent the mean ± SEM of three different replicates and are representative of at least three different experiments. ^*^P value < 0.05, **P value < 0.01, ***P value < 0.001.

The combination of 10 nM YM155 and 5 μΜ TPGS decreased the mRNA and protein levels of Survivin in SKBR3 cells to a larger extend than each agent alone (Fig. 1C). To investigate the mechanism of action, we examined the ability of the agents to induce apoptosis. Apoptotic induction was analyzed using the Alexa Fluor^TM^ 488 Dead Cell Apoptosis Kit, where Annexin V and PI are used for determination of apoptotic cell death. Treatment with 5 μΜ TPGS and 10 nM YM155 for 48 hours resulted in about 18% of SKBR3 staining positive for Annexin V (early apoptotic) and 30% for PI (late apoptotic) (Fig. 2A). The combination of agents did not induce an increase in the early or late apoptotic population of MCF-10A cells (Supplementary Fig. S3). TPGS did not enhance the effect of apoptotic effect of YM155 in MCF-7 cells (Supplementary Fig. S4), suggesting that the synergistic effect is cell-type specific.

To determine whether the growth inhibitory effect of the agents is accompanied by cell cycle arrest, we incubated cells with 5 μΜ TPGS and 10 nM YM155 for 48 hours and performed cell cycle analysis. The test agents, when administered either alone or in combination did not induce cell cycle arrest, but rather caused an increase in the subG1 phase (12.1% in the presence of YM155 alone and 22.1% with both agents) further supporting their ability to induce apoptosis in this cell line (Fig. 2B). DNA fragmentation was also detected using an Elisa-based assay. Treatment of SKBR3 cells with 5 μΜ TPGS + 5 nM YM155 increased DNA fragmentation 3-fold compared to 5 nM YM155 alone, and 6-fold compared to control. Much higher increase was evident when TPGS was combined with 7.5 nM YM155 (Fig. 2C).

**Figure 3:**
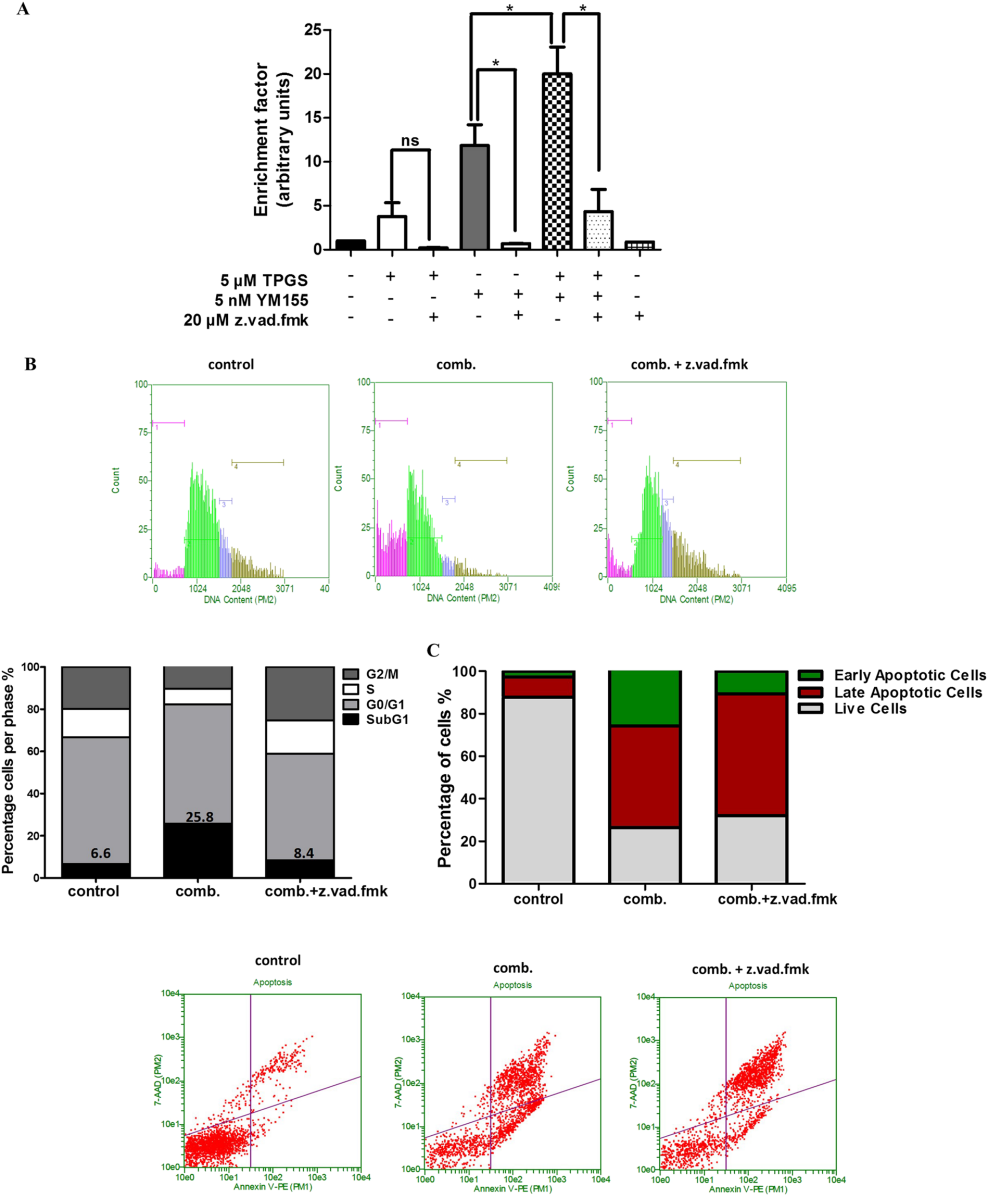
Reduction of DNA fragmentation caused by the combination of agents by z.vad.fmk. (**A**) Elisa-based DNA fragmentation detection in SKBR3 cells treated with 5 nM YM155, 5 μΜ TPGS with or without 20 μΜ z.vad.fmk for 48 hours. (**B**) Cell cycle analysis and (**C**) Annexin V/PI staining of SKBR3 cells treated with the combination of 5μΜ TPGS and 10 nM YM155 for 48 hours with or without z.vad.fmk. The results represent the mean ± SEM of three different replicates and are representative of at least three different experiments. ^*^P value < 0.05, **P value < 0.01, ***P value < 0.001.

DNA fragmentation was greatly reduced in the presence of the caspase inhibitor z.vad.fmk (Fig. 3A,B). The latter indicates the activation of caspase-dependent pathways by the combination of agents. However, even though co-incubation of TPGS and YM155 with z.vad.fmk reduced DNA fragmentation it did not restore the viability of cells (Figs 3C and S2) suggesting that caspase-independent pathways of cell death may also be activated. The supernatant of treated cells was also collected and evaluated for the presence of DNA fragments to detect necrosis. The supernatant of treated cells in the presence of the agents was not significantly enriched with DNA fragments suggesting that necrosis is likely not involved in their mode of action (Supplementary Fig. S5). In addition, treatment of SKBR3 cells with 5 and 10 nM YM155 alone or in combination with 5 μΜ TPGS did not increase the intracellular Reactive Oxygen Species (ROS) levels (Supplementary Fig. S5), suggesting that the induced DNA damage is not attributed to oxidative stress.

### YM155 and TPGS induce the intrinsic pathway of apoptosis in SKBR3 cells

**Figure 4:**
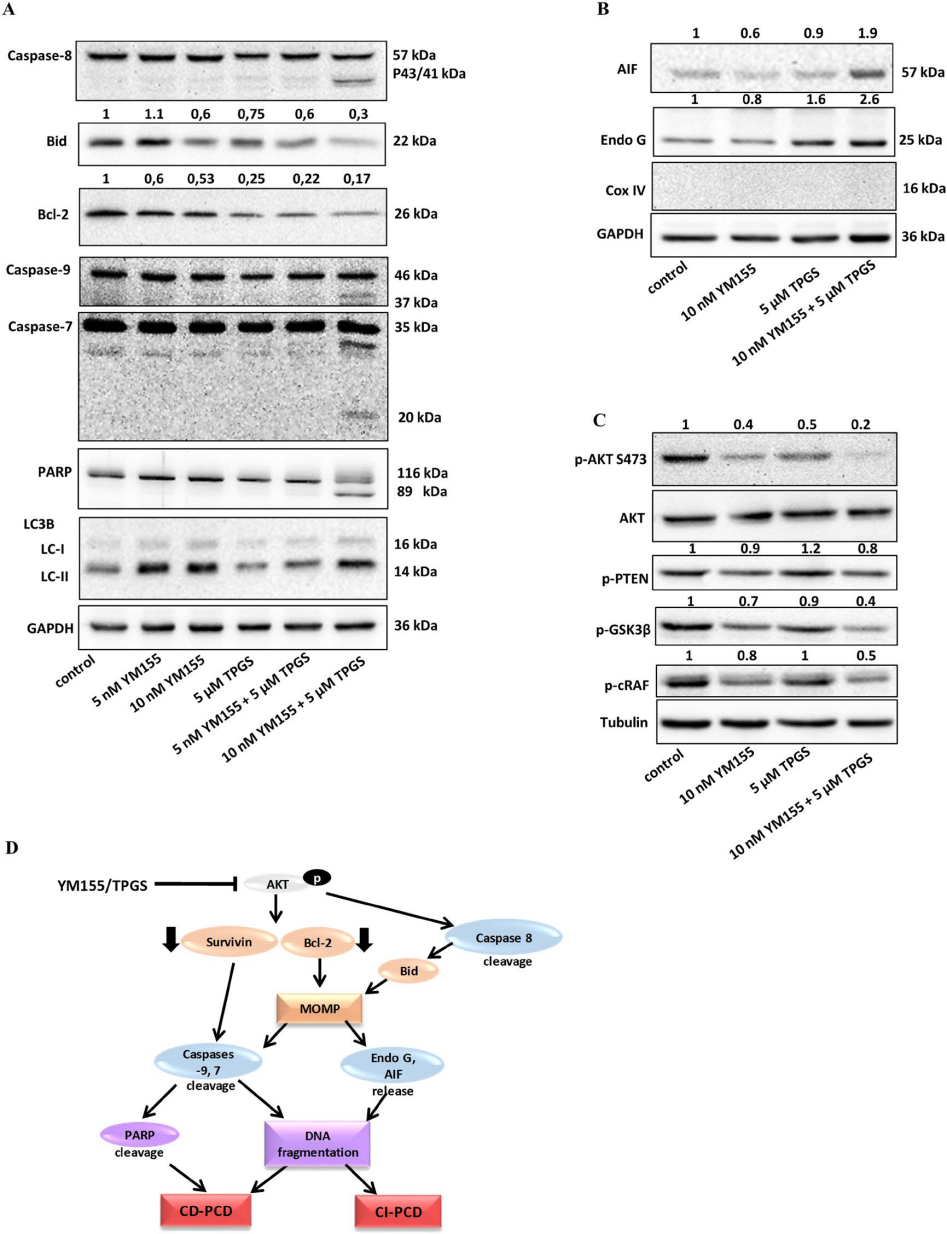
Effect of TPGS and YM155 on the levels and localization of apoptotic proteins. (**A**) The combination of 5 μΜ TPGS and 10 nM YM155 induces Caspase 8, 9, 7 and PARP cleavage and reduces the levels of Bcl-2 and Bid following 48 hours of treatment. YM155 alone produces the appearance of the LC-II protein (the blots were cropped; full length blots are shown in Fig. S7), (**B**) The combination of agents increased the levels of Endo G and AIF in the cytoplasm (the blots were cropped; full length blots are shown in Fig. S8), (**C**) The compounds inhibit the phosphorylation of AKT. SKBR3 cells were serum starved for 24 h, followed by incubation with 5 μM TPGS and 10 nM YM155 for 3 h and treated with 1 nM insulin, 1 hour prior to protein extraction (the blots were cropped; full length blots are shown in Fig. S9). (**D**) Potential mechanism of action of TPGS and YM155 in breast cancer. The agents inactivate the AKT pathway leading to the downregulation of Survivin and Bcl-2 and activation of Caspase-8. MOMP is increased causing Caspase 9 and 7 cleavage and release of Endo G and AIF to the cytosol, which lead to the activation of caspase-dependent and caspase-independent PCD. The results represent the mean ± SEM of three different replicates and are representative of at least three different experiments, ^*^P value < 0.05, **P value < 0.01, ***P value < 0.001.

To investigate the apoptotic pathway induced by the combination of agents, we determined their ability to affect the status of proteins involved in mitochondrial apoptosis. TPGS and YM155 reduced the levels of anti-apoptotic Bcl-2, a known inhibitor of the Mitochondrial Outer Membrane Permeabilization (MOMP) process^20^ (Fig. 4A). Consistently, these agents induced cleavage of Caspase-9, a target of pro-apoptotic proteins released from the mitochondria, and Caspase-7, its downstream target. PARP, a known substrate of active caspase-7^21^ was also cleaved 48 hours following the combination treatment (Fig. 4A). In addition, the combination of agents produced cleavage of initiator caspase-8 and reduced the levels of full length Bid which further enhances MOMP and mitochondrial apoptosis^22^. In agreement with previous reports, YM155 increased the amount of LC3B-II (Fig. 4A), a widely used autophagosomal marker, in SKBR3 cells^23,24^. However, co-treatment with TPGS did not further enhance the appearance of the LC-II form. This result is consistent with the inability of the autophagy inhibitor^25^ Bafilomycin to rescue cell viability in the combination treatment (Supplementary Fig. S2). These results indicate that autophagy is not involved in this scheme. The reduction of viability induced by the combination of YM155 and TPGS was not reversed by z.vad.fmk, indicating that capsase-independent pathways are also involved in their mode of action. To further investigate this possibility we assessed the cytosolic and nuclear levels of Apoptosis Inducing Factor (AIF) and Endo-G endonoucleases which have previously been shown to be involved in the induction of CI pathways of apoptosis^26^. Our results showed that the combination of agents caused an increase in the cytosolic levels of AIF and the 25 kDa form of Endo G providing strong support for the involvement of the caspace-independent apoptotic pathway (Fig. 4B).

### YM155 and TPGS inhibit the activation of the AKT pathway

AKT or Protein Kinase B (PKB), is often overactivated in cancer and plays a central role in the promotion of survival and inhibition of apoptosis. Akt is activated by phosphorylation within the carboxy terminus at Ser473^27^. Since the levels and activity of Survivin and Bcl-2 can be regulated via the PKB kinase^28,29^, we investigated the effects of the combination of agents on the phosphorylation status of the PI3K/AKT signaling pathway. YM155 and TPGS given as single agents reduced the levels of p-AKT at S473, while their combination almost abolished the phosphorylation of this residue (Fig. 4C). As a downstream effect, the levels of p-GSK3β, whose activity is inhibited by AKT-mediated phosphorylation at Ser9^30^, are also reduced. Phosphorylation of c-Raf by Akt at S259, which was also blocked by the TPGS-YM155 concurrent treatment, inhibits activation of the Raf-MEK-ERK signalling pathway and shifts the cellular response from cell cycle arrest to proliferation in human breast cancer cells^31^. The phosphorylation status of PTEN, a major upstream regulator of the PI3K/Akt signaling pathway, was not significantly affected in the presence of the agents (Fig. 4C).

## Discussion

Overexpression of Survivin has been reported in almost all human malignancies, including breast cancer, where it induces genetic instability^32,33^. The resulting population is resistant to apoptosis due to the overexpression of this IAP despite having extensive DNA damage^34^. YM155 causes DNA damage and downregulates Survivin, re-sensitizing in this manner this population of abnormal cells to apoptosis^24^; therefore, it is considered a promising treatment option and is being extensively investigated in a variety of cancers^35–37^. However, the efficacy of YM155 is hindered by its non-favorable pharmacokinetic profile^38^. Furthermore, short-term administration of high concentration of YM155 in the blood causes cardiotoxicity as well as nephrotoxicity^39^. In an attempt to lower the concentration of YM155 and improve its bioavailability, synergistic approaches as well as novel drug delivery systems have been developed. YM155 has demonstrated synergistic antitumor activity in combination with taxanes in lung cancer and melanoma models^40,41^ and sensitized human non-small cell lung cancer (NSCLC) cells to platinum drugs cisplatin and carboplatin^42^. YM155 also displayed synergistic action in neuroblastoma in *in vitro* and *in vivo* models in combination with lapatinib; the effect was attributed to lapatinib-induced inhibition of the ABCB1 efflux transporter, which allowed prolonged and elevated cytotoxicity of YM155^43^.

Recent studies have highlighted TPGS as an ideal molecular biomaterial in combination studies due to its multi-functional nature and its documented synergistic effectiveness with anti-cancer drugs^44^. Mixed micelles composed of a pH-sensitive poly(ethylene glycol)-doxorubicin conjugate prodrug and TPGS showed enhanced efficacy in multidrug resistant MCF-7/ADR cells^45^. The addition of TPGS in a nanocarrier loaded with Doxorubicin increased the therapeutic efficacy of the resulting nanoparticles, while a TPGS derivative was found to act synergistically with Docetaxel to reduce the viability of MCF-7 cells^46,47^.

In this study, we showed that the combination of YM155 and TPGS acts synergistically in SKBR3 breast cancer cells by de-activating the AKT survival pathway and inducing mitochondrial apoptosis. We also determined that the concentration of YM155 producing the highest synergy with TPGS is achievable, and well tolerated, in adult patients^11^. Importantly, the combination of agents did not produce significant cytotoxicity in normal immortalized breast cells. The effect of the combination of agents was specific to the SKBR3 cells that express high levels of HER2neu and have wild type PI3K/AKT and P-glycoprotein (Supplementary Table S1). HER2neu expression strongly correlates with PI3K/AKT activation^48^^,^ which may support SKBR3 sensitivity to these agents. In addition, TPGS may block the activity of WT P-glycoprotein^15^ which is present in SKBR3 cells, thereby allowing for an enhanced effect of YM155, compared to the other cell lines (Fig. 1B). The sensitivity of SKBR3 to the combination of agents may also be attributed to the fact that they express the highest Survivin levels amongst the cell lines tested^19^. TPGS and YM155 act antagonistically in MCF-7, MCF-10A and MDA-MB-231 cells (Fig. 1B and Table 1); even though YM155 has been described as a Survivin suppressant, its mechanism of action was later challenged and may also act by inducing DNA damage and Mcl-1 depletion^49^. It is possible that the two agents compete for interaction with molecules implicated in yet unidentified mechanisms of action in cells lacking a functional HER2/PI3K/AKT/Survivin axis.

In agreement with our previous report, TPGS was found to be effective in inducing apoptosis in breast cancer cells by reducing Survivin levels via the AKT/PKB pathway, but had no significant effect in normal immortalized cells^19^. In addition, TPGS was shown to induce apoptosis in T cell acute lymphocytic leukemia cells (but not on human peripheral blood lymphocytes) in a dose dependent manner; TPGS increased the expression of Bax and Puma, reduced mitochondrial membrane potential, caused Caspase-3 cleavage, nuclear DNA fragmentation and cell cycle arrest in ALL cells^50^.

The combination of TPGS and YM155 inhibited the AKT pathway, reduced Survivin and Bcl-2 levels and induced cleavage of Caspase-8, causing translocation of AIF and Endo G to the cytosol resulting in cell death mediated through both caspase-dependent and caspase-independent programmed cell death (PCD). Inhibition of p-AKT is known to cause Caspase-8 and Bid cleavage, thereby inducing MOMP^51^. Previous studies showed that YM155 induces caspase-8 dependent apoptosis, in human leukemia cells, through downregulation of Survivin and Mcl-1 and, in glioma cell lines to promote loss of mitochondrial membrane potential and release of AIF to the cytosol^52,53^. TPGS is known to activate both caspase-dependent and caspase-independent PCD in breast cancer cell lines, inducing the downregulation of Bcl-2, the translocation of AIF and Endo G to the cytosol and the cleavage of caspase-7 and PARP^19^. We propose here a mechanism of synergistic action between YM155 and TPGS, by which the combination of the two agents inhibits the phosphorylation of AKT and causes downregulation of Bcl-2 and Survivin followed by Caspase-8 cleavage. The mitochondrial pathway of apoptosis is initiated, allowing the activation of Caspases −9 and −7 and the release of AIF and Endo G to the cytoplasm which cause caspase-dependent and caspase-independent PCD respectively (Fig. 4D).

Since polymeric nanoparticles for the delivery of YM155 have been developed, our data suggest that the inclusion of YM155 in a TPGS-based nano-carrier may be an effective approach against breast cancer.

## Methods

### Cell cultures and reagents

SKBR3, MCF-7, MDA-MB-231, MDA-MB-361 and MCF-10A cell lines were obtained from the American Type Culture Collection (ATCC) (Manassas, VA). SKBR3 cells were cultured in McCoys media supplemented with 5% FBS and 1% antibiotic/antimycotic, MCF-7 and MDA-MB-231 cells in DMEM supplemented with 10% FBS and 1% antibiotic/antimycotic, MDA-MB-361 cells in DMEM-F12 supplemented with 10% FBS, 1% antibiotic/antimycotic and 4 mM L-glutamine and MCF-10A in DMEM-F12 supplemented with 20 ng/ml EGF, 100 ng/ml Cholera Toxin, 500 ng/ml Hydrocortizone, 10 μg/ml insulin, 5% Horse Serum (HS) and 1% Antibiotic, Antimycotic. DMEM, McCoys, FBS, HS, antibiotic/antimycotic and trypsin used in cell culture were purchased from Gibco, Invitrogen (Carlsbad, California, USA). PARP, AIF, p-AKT Ser476, total-AKT, p-PTEN, p-GSK3β, p-cRAF, Caspase -7, -8, -9 and Survivin antibodies were purchased from Cell Signaling Technology (Danvers, Massachusetts, USA). α-Τubulin antibody was purchased from Sigma (St. Louis, Missouri, USA). Bcl-2, GAPDH, Cox IV and Endo G antibodies were purchased from Santa Cruz Biotechnology Inc. LC3B antibody was purchased from Novus Biologicals Inc. (Littleton, CO, USA). YM155 and z.vad.fmk was obtained from Selleck chemicals (Houston, TX, USA). TPGS was purchased from Eastman Chemical Company (Kingsport, Tennessee, USA). All other reagents were purchased from Sigma (St. Louis, Missouri, USA).

### MTT assay

A total of 1 × 10^4^ cells were seeded per well of a 96-well plate and incubated for 24 hours. At the end of the incubation period, cells were treated with different concentrations of YM155 or (and where stated) in the presence of TPGS, for the time periods and concentrations described in the figure legends. The assay was performed as described elsewhere^54^. Cell viability was measured across a range of dose levels for the drug pair without maintaining the ratio of dose levels constant. The combination index (CI) was used for the quantification of synergistic, antagonistic or additive effects of the drug pair as described previously^55^. Calculation of the CI was performed using the CompuSyn program, version 1.0.

### Cell cycle analysis

Cells were seeded at a concentration of 1 × 10^6^ cells per well of 10 mm plate. Following incubation, samples were prepared as previously described^56^ and analyzed for DNA content using the Guava EasyCyte™ flow cytometer and the GuavaSoft analysis software (Millipore, Watford,UK).

### Cell death detection ELISA

Cells were seeded at a concentration of 1 × 10^4^ cells per well of a 96-well plate and incubated for 24 hours. Cells were treated with TPGS or YM155 as indicated. The quantification of mono- and oligonucleosomes present in the cytoplasm of apoptotic cells was performed using the Cell Death Elisa^PLUS^ Apoptosis Kit according to the manufacturer’s instructions (Roche).

### Annexin V/Propidium iodide staining

Cells were seeded at a concentration of 1 × 10^5^ cells per well of a 60-mm plate and treated with TPGS or YM155 as indicated. Cells were harvested and stained as described by Alexa Fluor^TM^ 488 Annexin V/Dead Cell Apoptosis kit (Life Technologies). Cell viability, death and apoptosis were evaluated using the Guava EasyCyte™ flow cytometer and the GuavaSoft analysis software (Millipore, Watford,UK). The annexin-V positive/PI negative cells were recognized as early apoptotic cells by the cytometer software whereas the annexin V positive/PI positive cells were identified as late apoptotic/dead cells. Similarly, the annexin V-negative/PI negative cells were identified as viable cells.

### Western blot analysis

To determine protein levels we performed Western blot analysis as described previously^18^. For preparation of mitochondrial and cytosolic extracts, the Mitochondria/Cytosol Fractionation Kit (ab65320) was used according to the manufacturer’s instructions (Abcam). The intensity values from the densitometry analysis of Western blots were normalized against GAPDH or α-Tubulin using ImageJ analysis software. Intensity values were expressed as fold change compared to control.

### Total RNA preparation and real-time quantitative PCR (q-PCR)

Total RNA was extracted with Trizol reagent (Invitrogen, Carlsbad, CA, USA) following the manufacturer’s protocol. cDNA was synthesized with random primers using the Superscript III Reverse Transcriptase (Invitrogen, Carlsbad, CA, USA). Primer sequences were designed using Primer3 and are as follows: human survivin, 5′-GACGACCCCATAGAGGAACA-3′(forward) and 5′-GACAGAAAGGAAAGCGCAAC-3′(reverse); and human GAPDH, 5´-TTGGTATCGTGGAAGGACTCA-3´(forward), 5´-TGTCATCATATTTGGCAGGTTT-3´ (reverse). Real-Time PCR was performed using the BioRad CFX96 Real-Time System and the SYBR Green PCR Master Mix (Applied Biosystems) according to the manufacturer’s instructions. The PCR products were normalized to those obtained from GAPDH mRNA amplification.

### Statistical analysis

Results for continuous variables were presented as Mean ± Standard Error. Two-group differences in continuous variables were assessed by the unpaired T-test. P-values are two-tailed with confidence intervals 95%. Statistical analysis was performed by comparing treated samples with untreated control. All statistical tests were conducted using Prism software version 5.0 (Graphpad, San Diego, California, USA).

## Supplementary information

Supplementary results and methods

## Data Availability

All materials, data and associated protocols mentioned in this manuscript will be available upon request.
