# Supplementary material for: d-a-Tocopheryl Polyethylene Glycol 1000 Succinate and a small-molecule Survivin suppressant synergistically induce apoptosis in SKBR3 breast cancer cells
Source: Sci Rep. 2019 Oct 7;9:14375. doi: 10.1038/s41598-019-50884-9 (PMC6779903; doi:10.1038/s41598-019-50884-9)
Supplement: Supplementary file 1 — Supplementary results and methods [file 41598_2019_50884_MOESM1_ESM.pdf]

Supporting Information

d-a-Tocopheryl Polyethylene Glycol 1000 Succinate and a small-molecule Survivin suppressant synergistically induce apoptosis in SKBR3 breast cancer cells

Christiana M. Neophytou[1], Avgoustinos Mesaritis[1], Gregoria Gregoriou[1] and Andreas I. Constantinou[1]*

[1] Department of Biological Sciences, Faculty of Pure and Applied Sciences, University of Cyprus, 1678, Nicosia, Cyprus, E-mail: cneoph01@ucy.ac.cy, mesaritis.avgoustinos@ucy.ac.cy, gregoriou.gregoria@ucy.ac.cy

*****Correspondence: Andreas I. Constantinou, Laboratory of Cancer Biology and Chemoprevention, Department of Biological Sciences, Faculty of Pure and Applied Sciences, University of Cyprus, P.O.Box 20537, 1678 Nicosia, Cyprus. E-mail: andreasc@ucy.ac.cy

Tel. No.: 00-35722-892883

Fax No.: 00-35722-892881

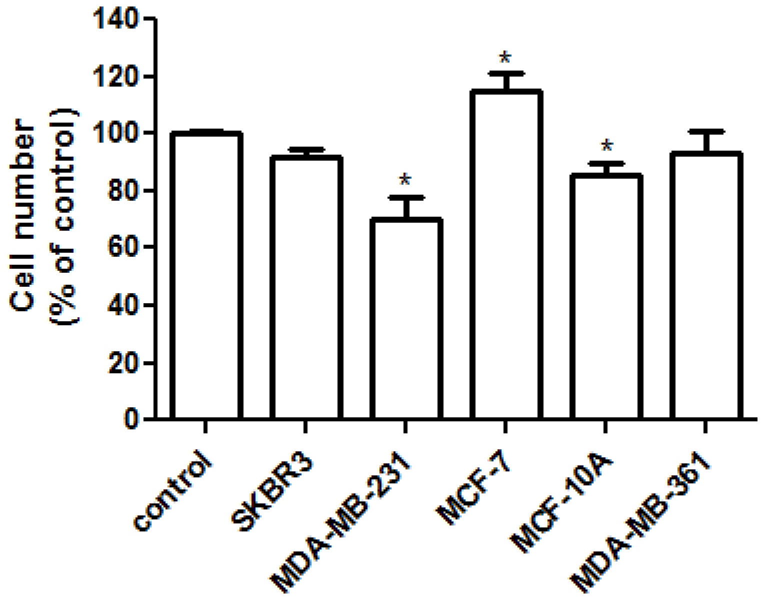

Supplementary Figure S1. Effect of TPGS on proliferation of breast cell lines. MTT assay was employed for the cytotoxicity evaluation (% cell viability) of 5 µM TPGS for 48 hours in SKBR3, MDA-MB-231, MCF-7, MDA-MB-361 and MCF-10A cells. The results represent the mean ± SEM of three different replicates and are representative of at least three different experiments. ∗P value < 0.05, ∗∗P value < 0.01, ∗∗∗P value < 0.001.

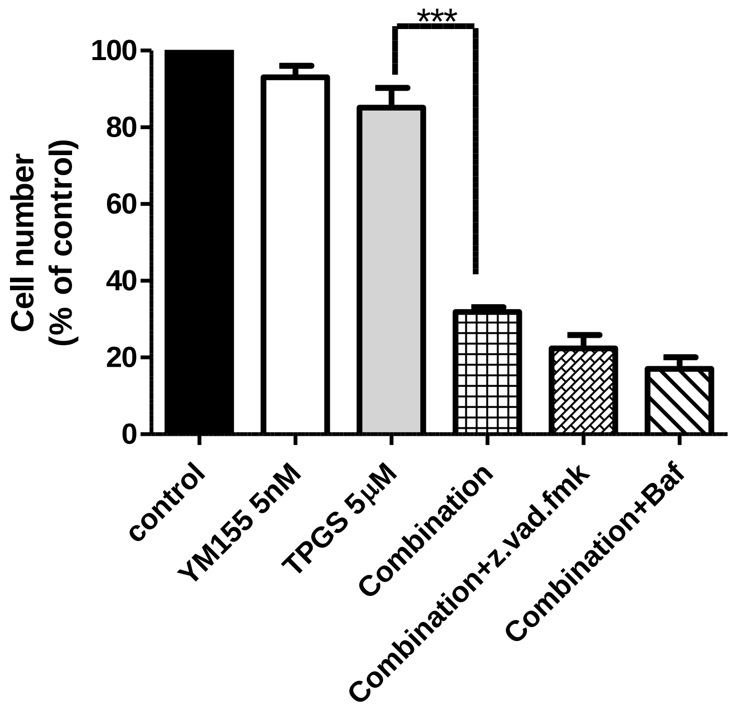

Supplementary Figure S2. Effect of inhibitors on the action of TPGS and YM155. Addition of 20 µM of z.vad.fmk or 100 nM Bafilomycine (Baf) did not restore the viability of SKBR3 cells when treated with the combination of YM155 and TPGS for 48 hours. The results represent the mean ± SEM of three different replicates and are representative of at least three different experiments. ∗P value < 0.05, ∗∗P value < 0.01, ∗∗∗P value < 0.001.

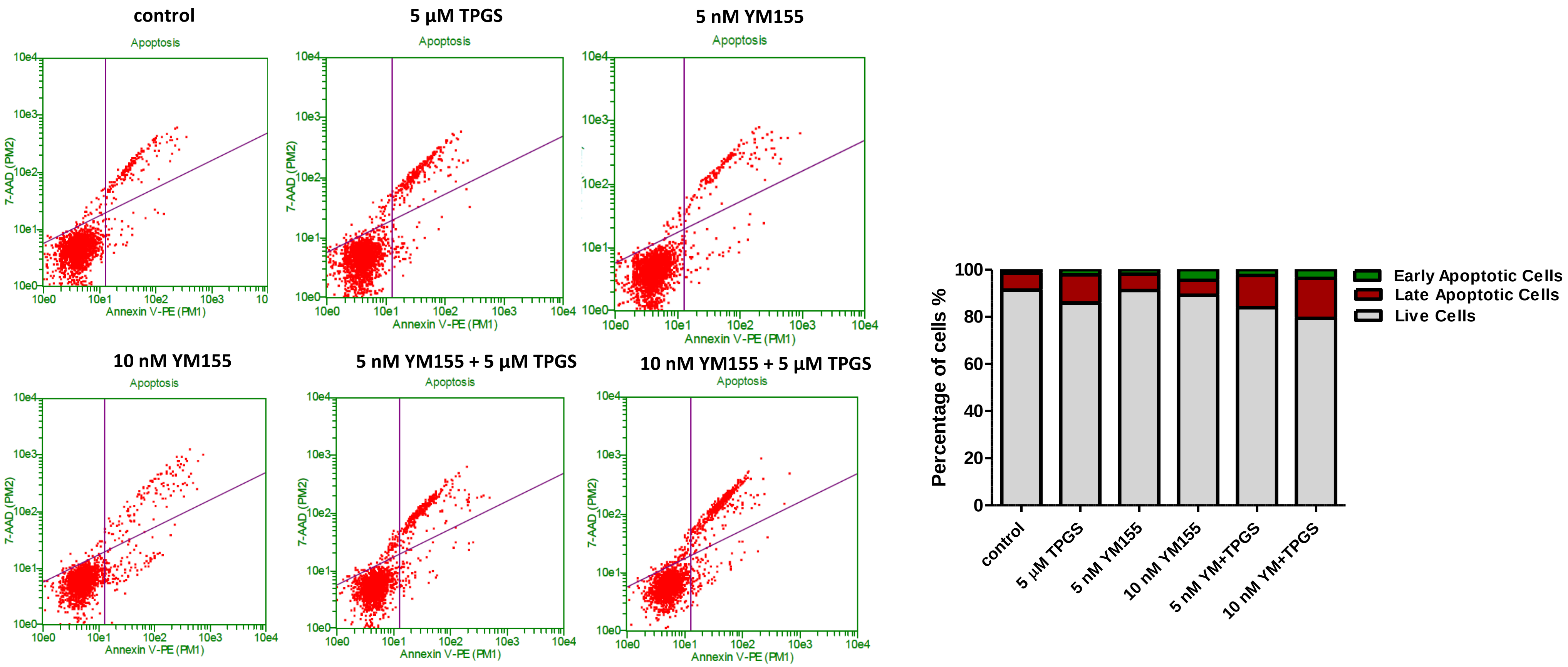

Supplementary Figure S3. Apoptotic effect of YM155 in combination with TPGS in MCF-10A cells. Annexin V/PI staining was employed for evaluation of the apoptotic effect (% compared to control) of increasing concentrations of 5 and 10 nM YM155 with or without 5 µM TPGS for 48 hours. The results are representative of at least three different experiments.

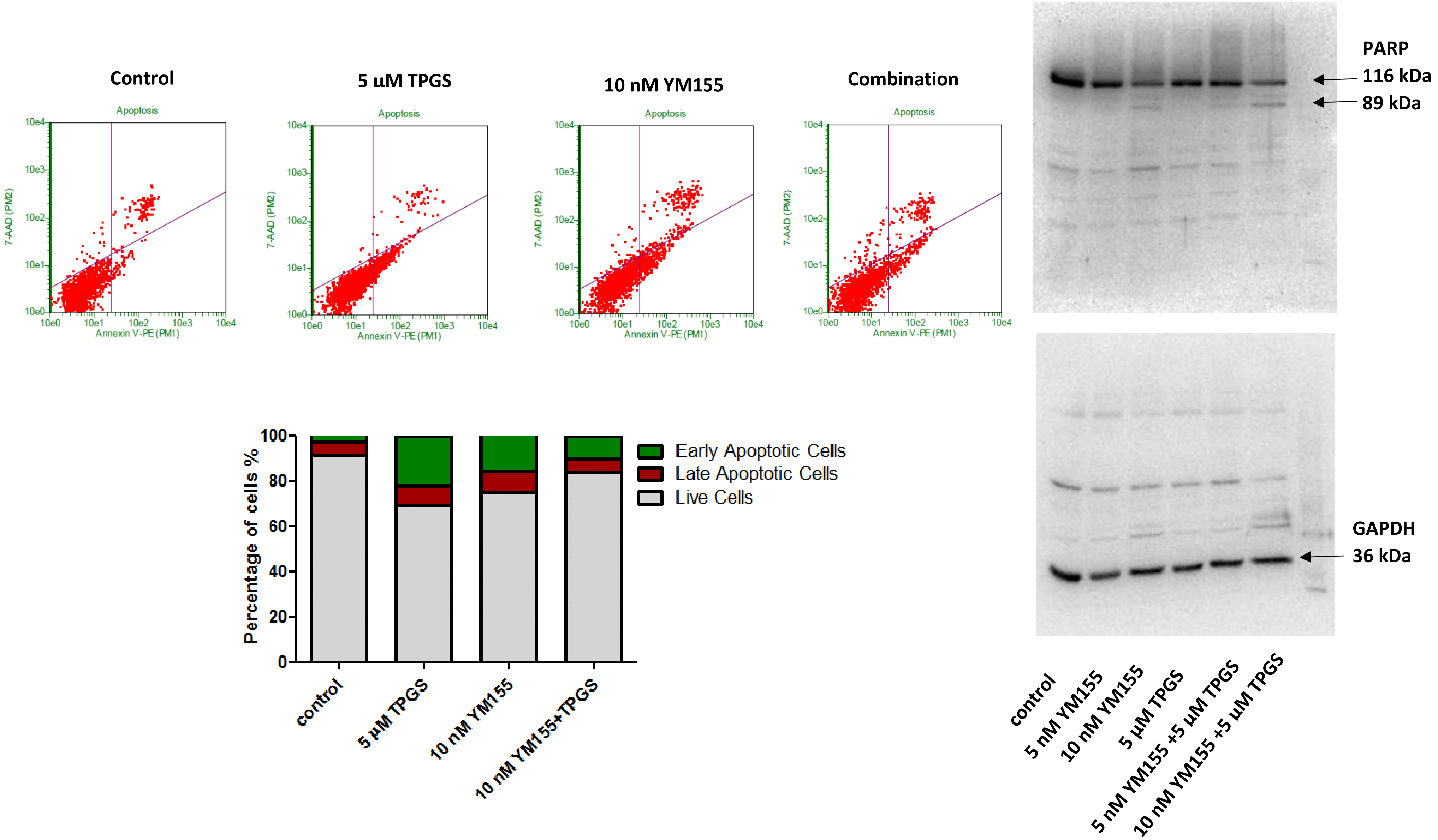

Supplementary Figure S4. Apoptotic effect of YM155 in combination with TPGS in MCF-7 cells. Annexin V/PI staining was employed for evaluation of the apoptotic effect (% compared to control) of 10 nM YM155 with or without 5 µM TPGS for 48 hours. Western Blot analysis shows that YM155 alone (at 10 nM) produces the appearance of the cleaved 89 kDa PARP protein; addition of 5 µM TPGS does not enhance this effect. The results are representative of at least three different experiments.

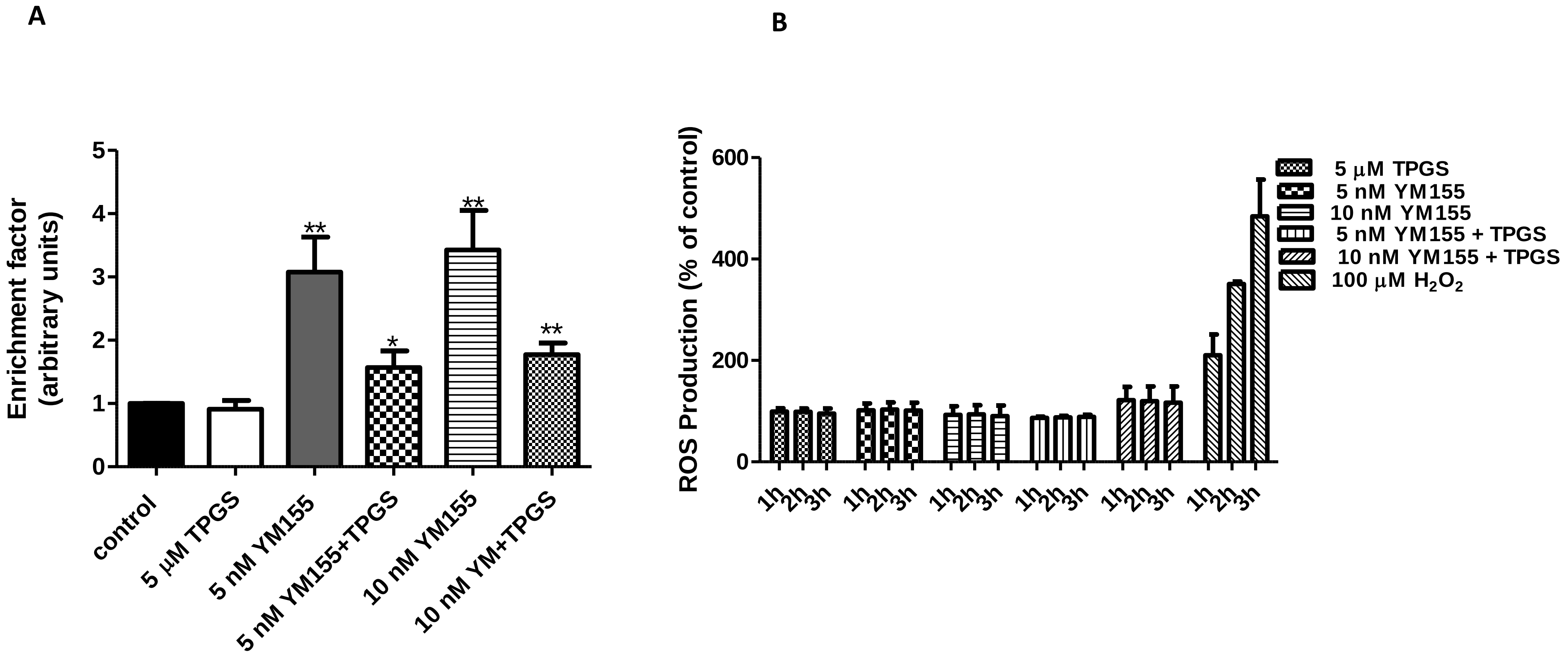

Supplementary Figure S5. The combination of agents does not enhance necrosis or induce production of reactive oxygen species. **A)** Elisa-based DNA fragmentation detection in the supernatant of SKBR3 cells treated with 5 or 10 nM YM155 alone or in combination with 5 µM TPGS at 48 hours. **B)** Time depended measurement of the production of ROS in SKBR3 cells in the presence of the agents. H_2O_2 at 100 µM was used as positive control. The results represent the mean ± SEM of three different replicates and are representative of at least three different experiments. ∗P value < 0.05, ∗∗P value < 0.01, ∗∗∗P value < 0.001

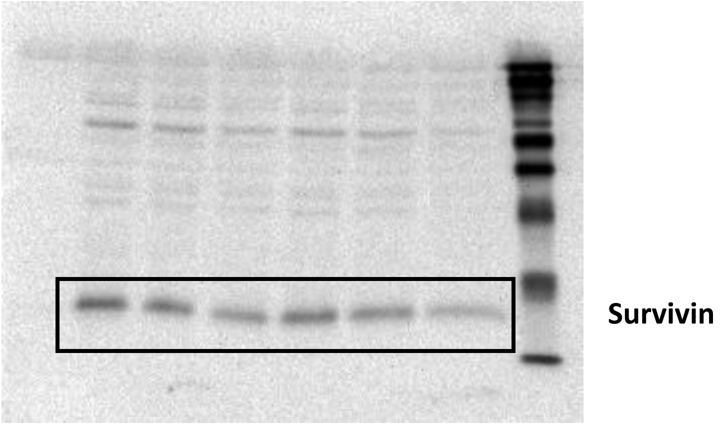

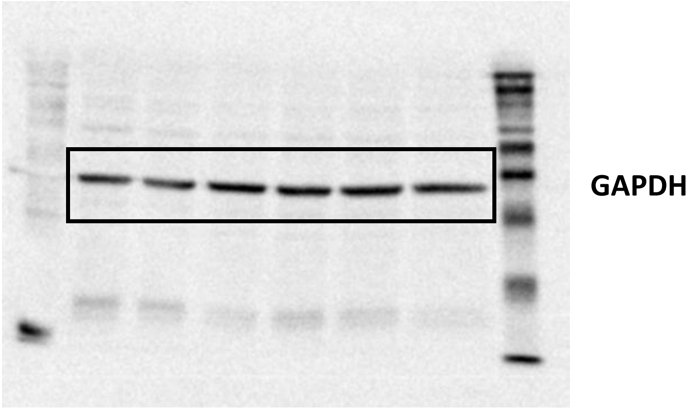

Supplementary Figure S6. The uncropped blots of Figure 1C. The black boxes indicate which bands are shown in the main paper.

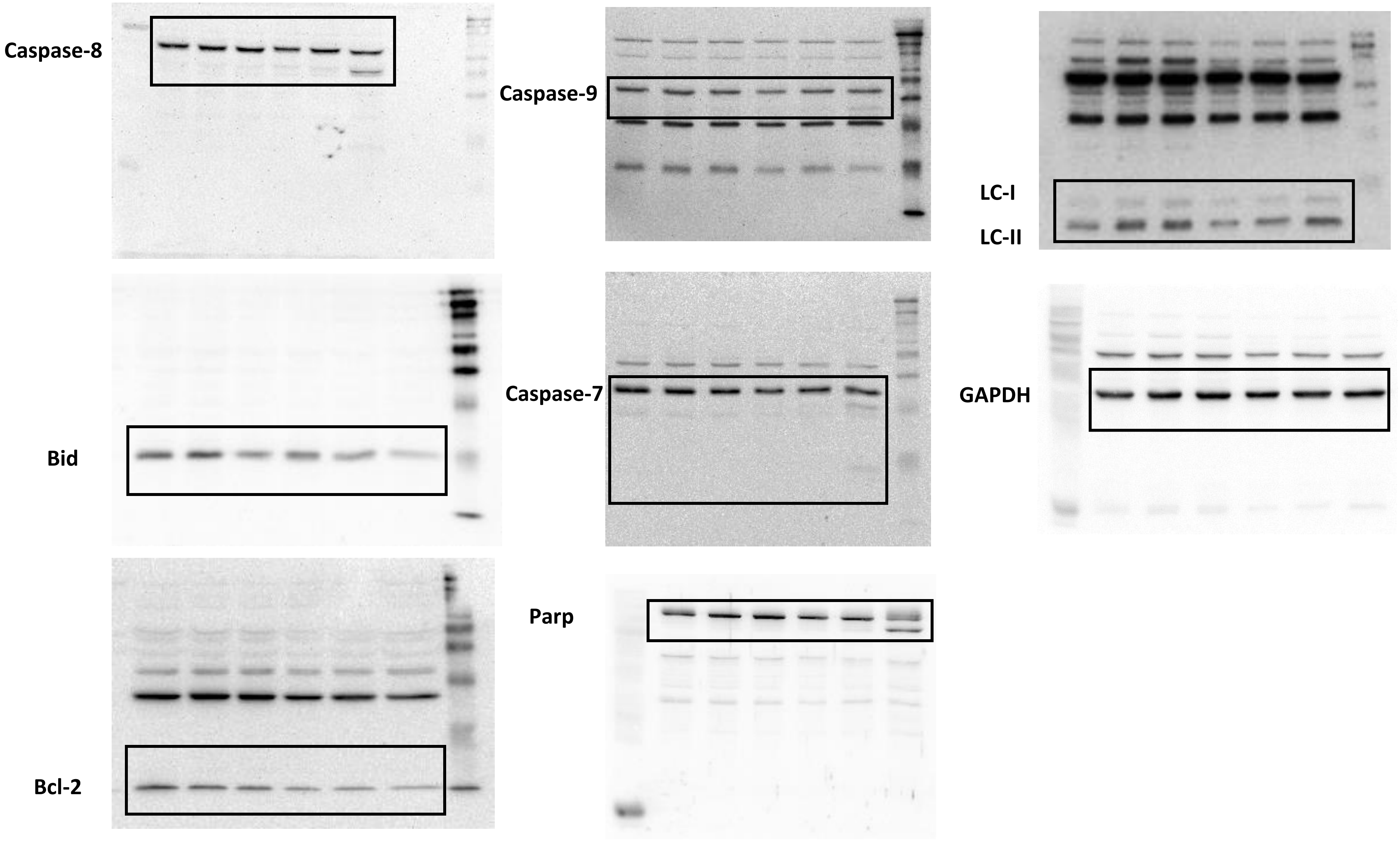

Supplementary Figure S7. The uncropped blots of Figure 4A. The black boxes indicate which bands are shown in the main paper. In the case of Caspases-9 and -7, high contrast was used in the main paper in order to make the bands of interest (cleaved caspases) adequately visible. The blots are displayed here at a lower exposure.

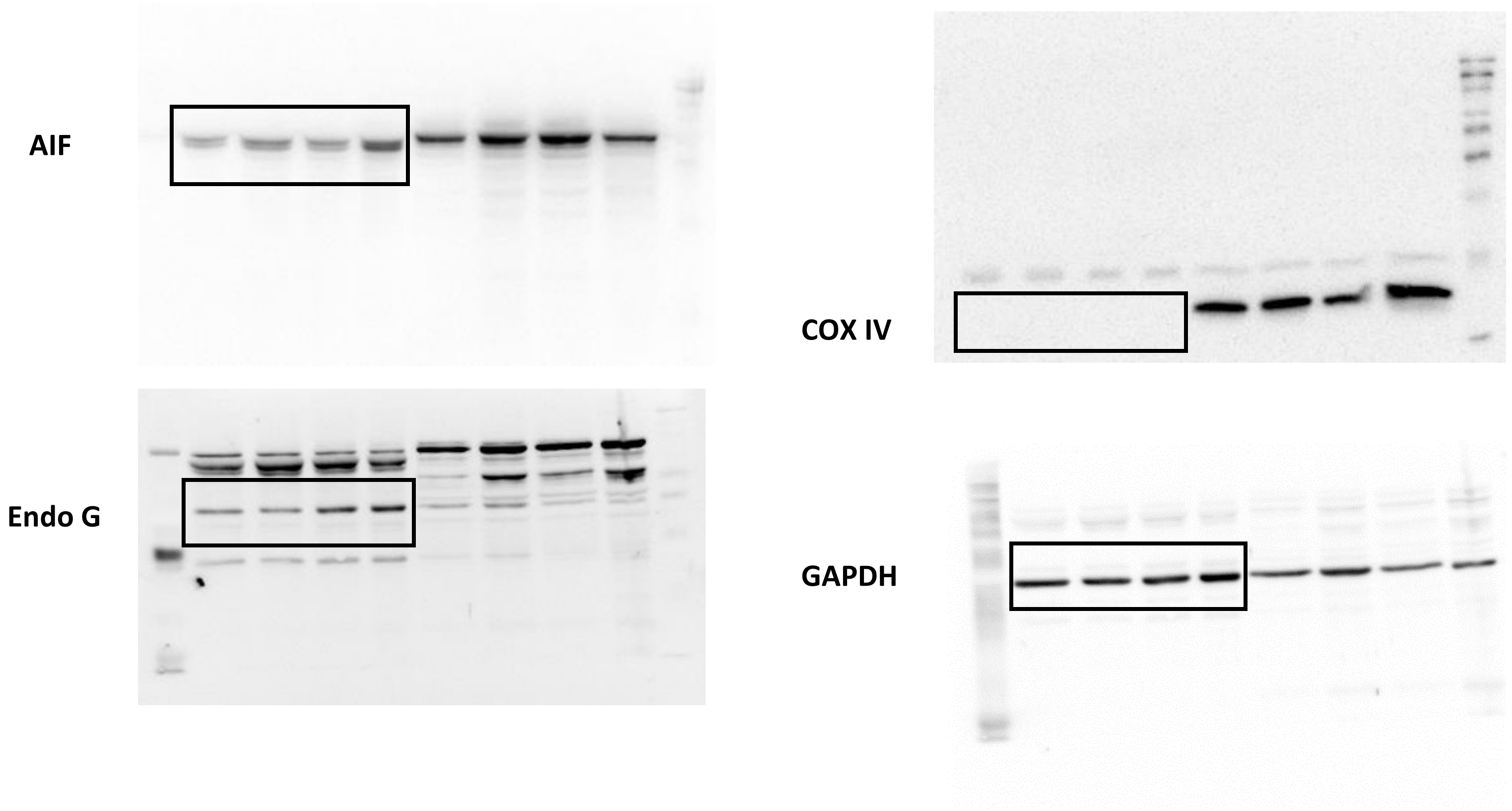

Supplementary Figure S8. The uncropped blots of Figure 4B. The black boxes indicate which bands are shown in the main paper.

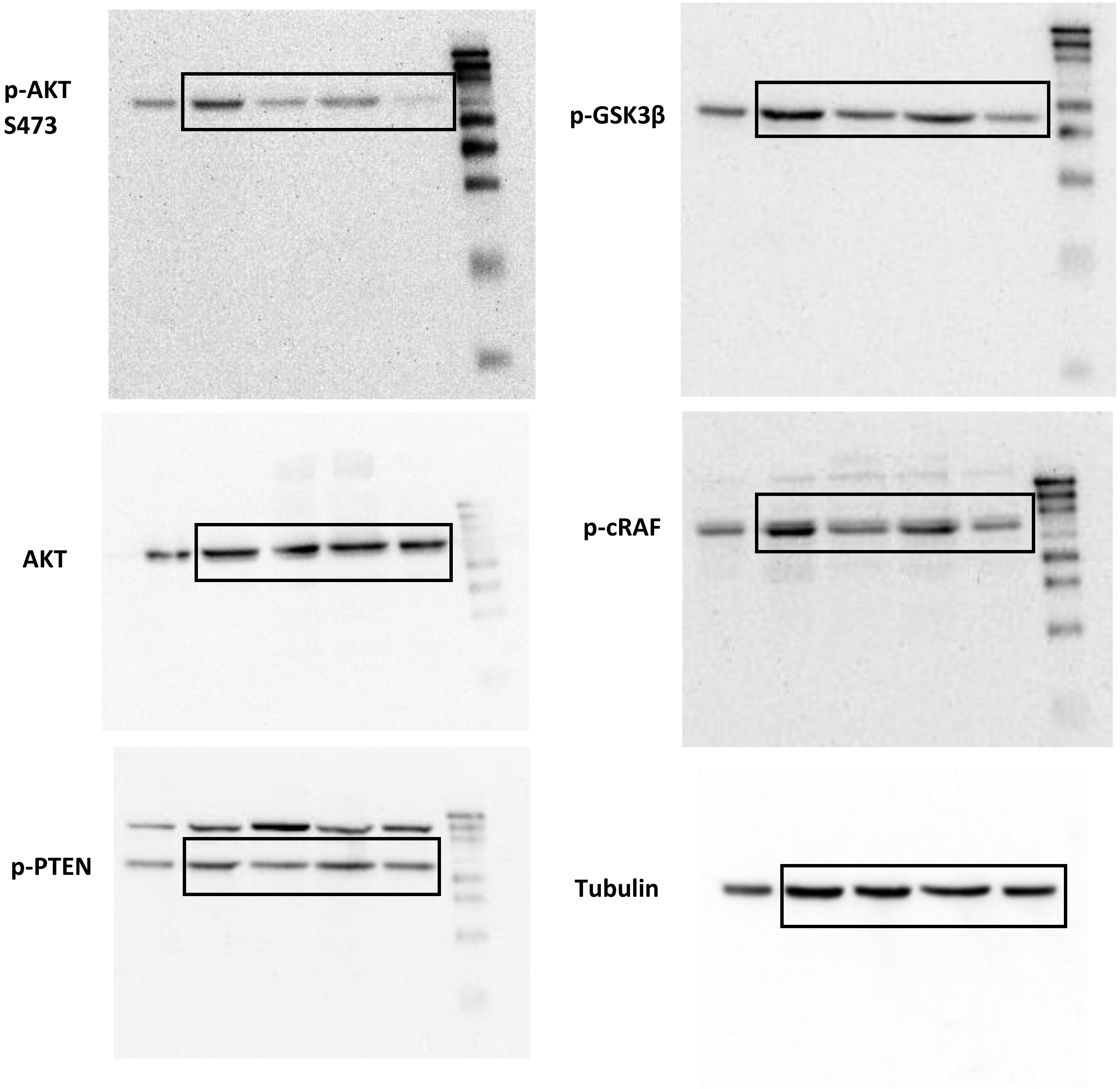

Supplementary Figure S9. The uncropped blots of Figure 4C. The black boxes indicate which bands are shown in the main paper.

Cell line	Subtype	Her2	PR	ER	P-gl	TP53	PI3K/AKT	Source	Tumor Type
**SKBR3***	**HER2**	**+**	**-**	**-**	**WT**	**M**	**WT/WT**	**PE**	**AC**
**MDA-MB-231**	**BaB**	**-**	**-**	**-**	**WT**	**M**	**WT/WT**	**PE**	**AC**
**MDA-MB-361**	**LuB**	**+**	**-**	**+**	**M**	**M**	**M/M**	**P.Br**	**AC**
**MCF-7**	**LuA**	**-**	**+**	**+**	**WT**	**WT**	**M/WT**	**PE**	**IDC**
**MCF-10A****	**BaB**	**-**	**-**	**-**	**-**	**WT**	**WT/WT**	**epithelial**	**non-tumorigenic**

Supplementary Table S1. Molecular classification of human breast cancer cell lines. Abbreviations: ER, estrogen receptor; PR, progesterone receptor; HER2, human epidermal growth factor receptor 2; P-gp, P-glycoprotein; WT, Wild Type; M, Mutated; AC, adenocarcinoma; BaB, basal B; IDC, invasive ductal carcinoma; Lu, luminal; P.Br, primary breast; PE, pleural effusion. Obtained from the COSMIC website unless otherwise indicated. *SKBR3 information obtained from [1, 2, 3, 4, 5]; **MCF-10A information derived from [6, 7].

1. Subik, K. et al. The Expression Patterns of ER, PR, HER2, CK5/6, EGFR, Ki-67 and AR by Immunohistochemical Analysis in Breast Cancer Cell Lines. Breast Cancer (Auckl) 4, 35-41 (2010).
2. Faneyte, I.F., Kristel, P.M. & van de Vijver, M.J. Determining MDR1/P-glycoprotein expression in breast cancer. Int J Cancer 93, 114-122 (2001).
3. Papanikolaou, V., et al., The involvement of HER2 and p53 status in the regulation of telomerase in irradiated breast cancer cells. Int J Oncol, 2009. 35(5): p. 1141-9.
4. She, Q.B., et al., Breast tumor cells with PI3K mutation or HER2 amplification are selectively addicted to Akt signaling. PLoS One, 2008. 3(8): p. e3065.
5. Liu, Z., X. Yu, and Z.A. Shaikh, Rapid activation of ERK1/2 and AKT in human breast cancer cells by cadmium. Toxicol Appl Pharmacol, 2008. 228(3): p. 286-94.
6. Yuan, T.L., et al., Cell-to-cell variability in PI3K protein level regulates PI3K-AKT pathway activity in cell populations. Curr Biol, 2011. 21(3): p. 173-83.
7. Peng, Z., et al., Dichotomy effects of Akt signaling in breast cancer. Mol Cancer, 2012. 11: p. 61

Supplementary Methods

ROS production assay

Cells are seeded at a concentration of 4 x 10^4 cells/ml in 12 well plates. Following 24 hours, media is removed and cells are treated with 100 µM DCFH-DA for 1 hour at room temperature in the dark. Media is removed and cells are washed twice with PBS. Fresh media containing the compounds under investigation is added. H_2O_2 at 100µM is used as positive control. Following treatment, fluorescence is measured at 1, 2 and 3 hours at excitation 485nm and emission 535nm.
